# Suitability of Cell-Based Label-Free Detection for Cytotoxicity Screening of Carbon Nanotubes

**DOI:** 10.1155/2013/564804

**Published:** 2013-11-27

**Authors:** Claudia Meindl, Markus Absenger, Eva Roblegg, Eleonore Fröhlich

**Affiliations:** ^1^Center for Medical Research, Medical University of Graz, 8010 Graz, Austria; ^2^Department of Pharmaceutical Technology, Institute of Pharmaceutical Sciences, Karl-Franzens-University of Graz, 8010 Graz, Austria

## Abstract

Cytotoxicity testing of nanoparticles (NPs) by conventional screening assays is often complicated by interference. Carbon nanotubes (CNTs) are particularly difficult to assess. To test the suitability of cell-based label-free techniques for this application, a panel of CNTs with different diameters and surface functionalizations was assessed by impedance-based technique (*xCELLigence* RTCA) and automated microscopy (Cell-IQ) compared to formazan bioreduction (MTS assay). For validation of the label-free systems different concentrations of ethanol and of amine (AMI) polystyrene NPs were used. CNTs were evaluated in various cell lines, but only endothelial EAhy926 cells and L929 and V79 fibroblasts could be evaluated in all systems. Polystyrene particles obtained similar results in all assays. All systems identified thin (<8 nm) CNTs as more cytotoxic than thick (>20 nm) CNTs, but detection by *xCELLigence* system was less sensitive to CNT-induced cytotoxicity. Despite advantages, such as continuous monitoring and more detailed analysis of cytotoxic effects, label-free techniques cannot be generally recommended for cytotoxicity screening of NPs.

## 1. Introduction

Nanoparticles (NPs) are used in a variety of industrial, consumer, and medical products. Their application field would even be much broader if the toxicological potential was better known. For the initial evaluation of compounds cytotoxicity testing by screening assays (CSAs) is of key importance. Conventional CSAs are based on the quantification of enzyme activity, protein content, DNA content, and organelle function. These detections are based on colorimetric, fluorometric, luminescent, and, less frequently, radiometric measurements. In contrast to conventional drug compounds, however, the assessment of NPs in these assays is more problematic since they can interfere at various levels with the detection. NPs can catalyse the conversion of tetrazolium salts [1–3], absorb dyes [4, 5], and interfere with absorbance [6, 7] and with fluorescence [5, 8]. They may also adsorb proteins [9], degrade indicator dyes [10], cause redox reactions [11], and interfere by light scattering [12, 13]. Carbon nanotubes (CNTs) belong to the NPs with the highest degree of interference with CSAs [1, 2, 4, 14]. Interference with assays appears to be particularly likely when the protocol affords lysis of the cells [15]. In this situation, testing by label-free techniques could be advantageous. Testing in the absence of dyes might also be important because influence of dyes on cellular function has been reported. 2′,7′-Bis(2-carboxyethyl)-5-(and 6)-carboxyfluorescein (BCECF-AM), used for measurement of intracellular pH, and rhodamine 6G, used for labelling of mitochondria, can dose-dependently block migration in phagocytes [16].

Label-free techniques used for cell viability include refractive index-based technologies, fibre optic waveguide measurements, acoustic technologies, impedance-based instruments, and automated microscopy. Refractive index-based technologies are particularly suitable to address receptor-mediated signalling. Fibre optic waveguide measurements are used for the detection of oxygen consumption as parameter for mitochondrial respiration and for extracellular acidification as indication for glycolysis. Acoustic technologies using resonant frequency of piezoelectric quartz crystals, impedance-based instruments, and automated microscopy are suitable for cytotoxicity testing.

Label-free CSAs have the additional advantage that they allow continuous monitoring. Continuous measurement in contrast to endpoint detection can identify potential cellular adaptations to the toxic compound. Usually, compounds decrease viability to greater extent after longer than after shorter exposure times (e.g., [17, 18]). Adaptation to toxic stimuli, however, has also been reported. Liver cells can adaptate by changes in enzyme activities like, for instance, hexokinase, phosphoenolpyruvate carboxykinase, cyclooxygenase 2, *γ*-glutamyl transferase, and various biotransformation enzymes [19]. Other mechanisms include induction of membrane glycoprotein, heat shock proteins, and multidrug resistance membrane pumps. While viability of HaCAT cells exposed to the same concentration of silver NPs decreased in the order 24 h-48 h-72 h, that of HeLa cells was also considerable lower at 48 h than at 24 h. However, almost no difference was seen between viabilities at 48 h and 72 h [20]. These changes are important to know since this stress reaction might work in normal cells but not in cells that are already under stress.

Although label-free CSAs could be ideal for cytotoxicity screening of NPs, CNTs can act as semiconductors and, therefore, can interfere with the measurements based on currents. On the other hand, measurements based on optical recognition could have problems in recognizing cells in the presence of black CNT precipitates.

In the following two label-free techniques were used, the impedance-based *xCELLigence* real time cell analyzer (RTCA) and the Cell-IQ Analyzer, based on automated microscopy. Impedance-based instruments use two gold electrodes, one sensor electrode beneath the cells and a counter electrode. An alternate current in the presence of electrolytes in the medium leads to the generation of an electric field, where the cellular plasma membrane acts as insulator. The covering of the sensor electrode with cells forces the current to pass between or under the cells and causes an increase in the impedance. Measurements by *xCELLigence* RTCA produced reliable results in the toxicological assessment of several metal oxide NPs (ZnO, CuO [21, 22]; SiO_2_ [21, 22]). These NPs, however, cause only low interference with screening assays because they do not show obvious colour or tendency for precipitation. Automated microscopy works with phase contrast and takes advantage of morphological changes in the cells. The cells can be located inside an incubator or as integrated platform. With this method a distinction of specific population of cells can be made. The classification into resting (stable) cells, dead cells, and dividing cells is common [23–25]. In addition, differentiated cells have been separated from nondifferentiated cells [26]. Although this technique has been employed for microscopical validation of the results, it has not been used for cytotoxicity testing.

To study the suitability of *xCELLigence* RTCA and Cell-IQ analyzer for the assessment of CNTs, cytotoxicity was assessed in different cell lines in both systems, in addition to evaluation by formazan bioreduction (MTS). For validation of the label-free systems, different concentrations of ethanol and 20 nm amine polystyrene (AMI) particles were used. Plain and carboxyl-functionalized short CNTs in various diameters were studied.

## 2. Materials and Methods

### 2.1. Cells

Short CNTs (0.5–2 *μ*m) with and without COOH-functionalization were purchased from CheapTubes Inc. (Brattleboro, Vermont). These CNTs are synthesized by catalytic chemical vapour deposition and are purified with dilute nitric acid. Multiwalled CNTs are functionalized through repeated reductions and extractions in KMnO_4_ (unpublished information from the provider) and show a low amount of contaminants (ash < 1.5 wt%). SCNTs contain <3 wt% amorphous carbon and 5-6 wt% DWCNTs/MCNTs (http://www.cheaptubes.com/shortcoohcnts.htm#ixzz1YPExQhZi). SCNTs are functionalized with air oxidation (unpublished information from the provider).

Single-walled plain and carboxylated CNTs (termed as SCNT and SCNTc) with 1-2 nm diameter, purity >90%, and multi-walled CNTs in the diameters <8 nm, 20–30 nm and >50 nm, purity >95%, termed as MCNT8 and MCNT8c, MCNT20 and MCNT20c, and MCNT50 and MCNT50c were used.

### 2.2. Physicochemical Characterization

20 nm amine polystyrene particles (Estapor, Roche) were characterized by dynamic light scattering using a Malvern Zetasizer 3000 HS (Malvern). Particles were diluted with DMEM + 10% FBS to 200 *μ*g/mL and sonicated for 20 min. After equilibration of the sample solution to 25°C, size and zeta potential were measured at 633 nm and a detection angle of 90°. NNLS software was used for sample analysis.

CNTs suspended in DMEM +10% fetal bovine serum have been characterized physicochemically by Dynamic Light Scattering and Laser Doppler Velocimetry (Nanosizer, determination of size and surface charge), transmission electron microscopy (FEI Tecnai G² 20, determination of size), and energy dispersive X-ray spectroscopy (FEI Tecnai G² 20 with Standard SUTW detector, detection of metal contamination) as described in a previous study [15]. Heavy metal contamination was shown to be below detection threshold. Diameters according to TEM were very close to the data indicated by the producer (Table 1). Lengths, however, were considerably shorter than indicated by the producer, presumably induced by the ultrasound treatment used to improve dispersion of the tubes in the medium. Surface charges of plain and carboxyl-functionalized CNTs were all slightly negative. As indicated by the producers, all CNTs have a medium conductivity of >100 S/cm (http://www.cheaptubes.com/shortcoohcnts.htm).

### 2.3. Cell Culture

DMBM-2 mouse macrophages, murine L929 and V79 Chinese hamster lung fibroblasts (Deutsche Sammlung für Mikroorganismen und Zellkulturen GmbH), endothelial EAhy926 cells (kind gift from Dr. Edgell), and human MRC-5 (ATCC) fibroblasts were studied. Cells were cultured and seeded 24 hours before treatment in the medium recommended by the provider. CNTs were applied to cells suspended in cell culture medium after sonication in an Elmasonic S40 water bath (ultrasonic frequency: 37 kHz, 40 W, Elma) for 20 min. Exposures were performed at 37°C in a 95% air/5%CO_2_ atmosphere.

### 2.4. Cytotoxicity by Formazan Bioreduction

Cells were seeded EAhy 926 cells: 1∗10^4^/well; L929, MRC-5, and V79: 1.25∗10^4^/well; DMBM-2: 3∗10^4^/well) and evaluated after 4 h, 24 h, and 48 h of exposure to particles and EtOH. CellTiter 96 AQueous Nonradioactive Cell Proliferation Assay (Promega) was used according to the manufacturer's instructions. Not ingested CNTs were removed by repeated washings with PBS. 20 *μ*L of the combined MTS/PMS solution was added to 100 *μ*L fresh medium in each well and plates were incubated for 2 hours at 37°C in the cell incubator. The supernatant was transferred to a new plate to ensure that the signal was not influenced by absorbance of CNTs incorporated into cells. For the testing of ethanol and 20 nm AMI particles, washing and transfer of the supernatant to a new plate were omitted and absorbance was read at 490 nm on a plate reader (SPECTRA MAX plus 384, Molecular Devices).

### 2.5. Cytotoxicity by Impedance Measurement

**Figure 1:**
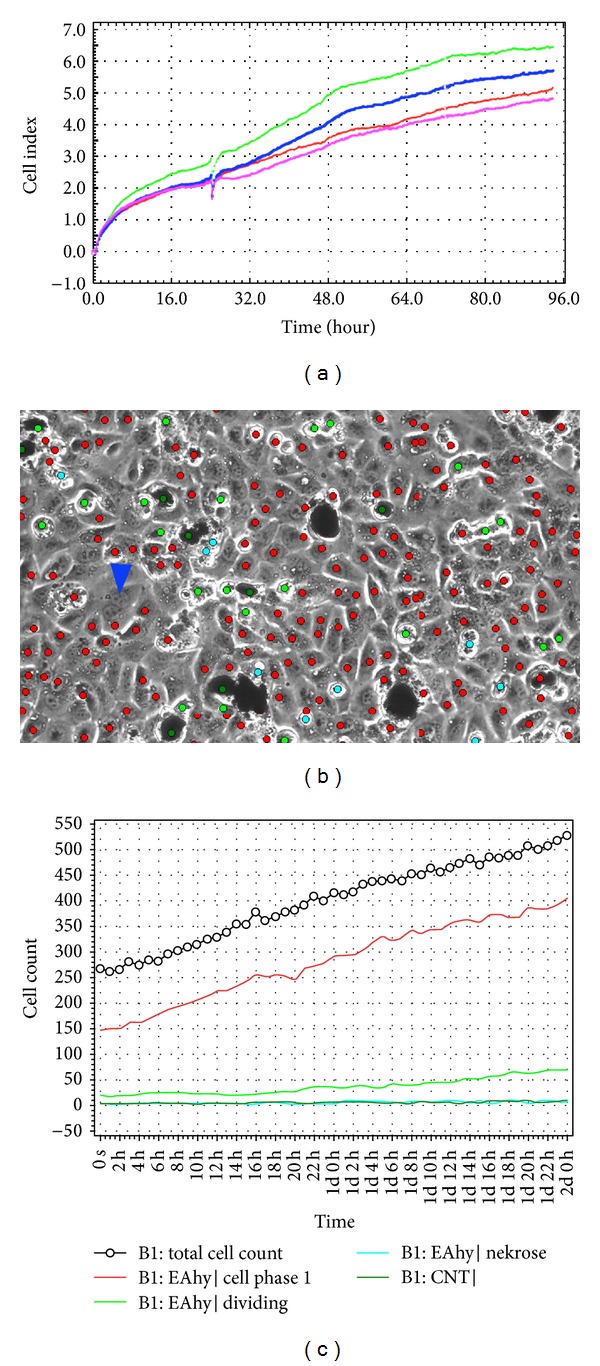
Raw data of *xCELLigence* and Cell-IQ analysis software. (a) Growth curve of EAhy cells, untreated (green and red) and in the presence of 25 *μ*g/mL MCNT20 (blue and pink). Image analysis by Cell-IQ software. (b) Classification of cells into stable (G1 cells, red), dividing cells (light green), necrotic cells (turquoise), and CNTs (dark green). Binucleated cells have to be identified manually (arrow head). (c) Time-related changes in the number of stable cells (red), dividing cells (light green), necrotic cells (turquoise), and CNTs (dark green) based on this classification are displayed in a graph.

Experiments were carried out using the *xCELLigence* RTCA DP instrument (Roche Diagnostics GmbH) which was placed in a humidified incubator at 37°C and 5% CO_2_. Experiments were performed using modified 16-well plates (E-plates, Roche Diagnostics GmbH). Microelectrodes were attached at the bottom of the wells for impedance-based detection and 100 *μ*L of cell-free growth medium was added to the wells. After leaving the devices at RT for 30 min, the background impedance for each well was measured. 50 *μ*L of the cell suspension containing 1∗10^4^ EAhy926 cells, 2∗10^4^ DMBM-2, 1.25∗10^4^ L929, and MRC-5 cells, and 1∗10^4^ V79 cells was seeded into the wells. Cell numbers that allowed optimum growth over the entire incubation period were determined for every cell line in pilot experiments. After leaving the plates at room temperature for 30 min to allow cell attachment, plated were transferred into the RTCA DP device and impedance monitored by the *xCELLigence* system. After 24 h (when the cell index had reached at least 1.0), 50 *μ*L of nanoparticles suspended in medium or EtOH in medium was added. Each concentration was tested in duplicate within the same experiment. Cell index (CI) was monitored every 15 min for 48 h and data recorded by the supplied RTCA software (Figure 1(a)). For comparison with MTS data, raw data with high time resolution resulting from independent *xCELLigence* experiments were reduced to a lower time resolution by selecting only the data points corresponding with the time points of the MTS assay. Data were imported in Excel and CI-data normalized to untreated cultures.

### 2.6. Cell Proliferation and Morphology Studies

Cells were seeded onto 24-well plates (EAhy 926 cells: 1∗10^5^/well; fibroblasts L929, MRC-5, and V79: 5∗10^4^/well; DMBM-2: 2∗10^5^/well) prior to the exposur. After addition of nanoparticles or EtOH in medium, cells were monitored with integrated optics for phase contrast imaging and machine vision technology in a humidified, 5% CO_2_ atmosphere at 37°C for 48 h (Cell-IQ, Chip-man Technologies Ltd). Images were captured automatically from 4 positions per well at 1 h intervals. A protocol for examining cell numbers and morphology with Cell-IQ analyzer software was created according to manufacturer instructions. Briefly, segmentation parameters were first adjusted to recognize cells and classify them into necrotic, dividing, apoptotic, and stable (G1-phase) cells. In addition, a class was added for CNTs and cell debris + background (garbage). The protocol was tested and optimized by comparing software classification of a set of sample images with manual classification. The protocol was then used to analyse images taken during Cell-IQ experiments (Figure 1(b)). Data were exported to Microsoft Excel and analysed further. For comparison with MTS data, raw data with high time resolution resulting from independent Cell-IQ experiments were reduced to a lower time resolution by selecting only the data points corresponding with the time points of the MTS assay. First, garbage and CNTs were subtracted from total cell numbers (corrected total cell numbers). The numbers of resting, necrotic, and dividing cells were then normalized to 100 corrected total cells. This was necessary because each observation field contained different numbers of cells at the start. The normalized number in the respective class was then related to the number in the untreated cultures as 100%.

### 2.7. Statistical Analysis

Data from three to four independent experiments were subjected to statistical analysis. This data is represented as means ± S.D. data and has been analyzed with one-way analysis of variance (ANOVA), followed by a Tukey-HSD post hoc test for multiple comparisons (SPSS 19 software). Results with *P* values <0.05 were considered to be statistically significant.

## 3. Results

### 3.1. Physicochemical Characterization of CNTs

20 nm AMI particles were 54 nm large with a zeta potential (*ζ*) of −9.02 mV in the exposure medium, as characterized by Dynamic Light Scattering and Laser Doppler Velocimetry. CNTs, in addition to Dynamic Light Scattering, were also characterized by transmission electron microscopy and EDX analysis, as described earlier [15]. Diameters as determined by TEM were similar to what was indicated by the producer. Hydrodynamic diameters, with the exception of MCNT50, were 2-3 times larger than indicated. All CNTs possessed a slightly negative surface charge. Heavy metal contamination was below threshold. A summary of CNT parameters is presented in Table 1.

### 3.2. Suitability of Cell Lines for Label-Free Detection

Regulatory cytotoxicity testing of adherent cells is performed at the end of the logarithmic phase, at 80% confluency (EN/ISO 10993-5 guideline). Since the growth area of E-plates wells (0.2 cm^2^) is smaller than that of 96-well plates (0.32 cm^2^) and only one tenth of 24-well plate (2 cm^2^), different cell numbers had to be seeded. Seeding densities from the MTS assay were tested and potentially adapted in pilot experiments on the label-free systems in order to allow 48 h of recording in untreated cultures (Table 2).

While EAhy926 and L929 cells could be studied in all systems, DMBM-2 cells could not be assessed neither in *xCELLigence* system nor in Cell-IQ. MRC-5 could be analysed in the *xCELLigence* system but not in the Cell-IQ-system, while V79 were suitable for Cell-IQ but not for *xCELLigence* system.

The *xCELLigence* system worked best with endothelial cells. While DMBM-2 cells and V79 cells, independent from the seeding density, did not reach the required cell index of 1, EAhy 926 cells reached a cell index of 2.5 ± 0.5. The fibroblasts reached lower values of 1.7 ± 0.5 (MRC-5) and 1.3 ± 0.8 (L929). After 48 h the cell index of the EAhy926 cells had further increased to 3.9 ± 0.7, while it remained constant for the fibroblasts.

For the evaluation in the Cell-IQ, epithelioid shape was better than spindle shape. MRC-5 cells were too large to fit into the image window of the classification library, which made reliable cell classification impossible. Classification of V79 cells worked less reliably due to variation in the (patchy) growth pattern.

### 3.3. Assessment of Control Substances

**Figure 2:**
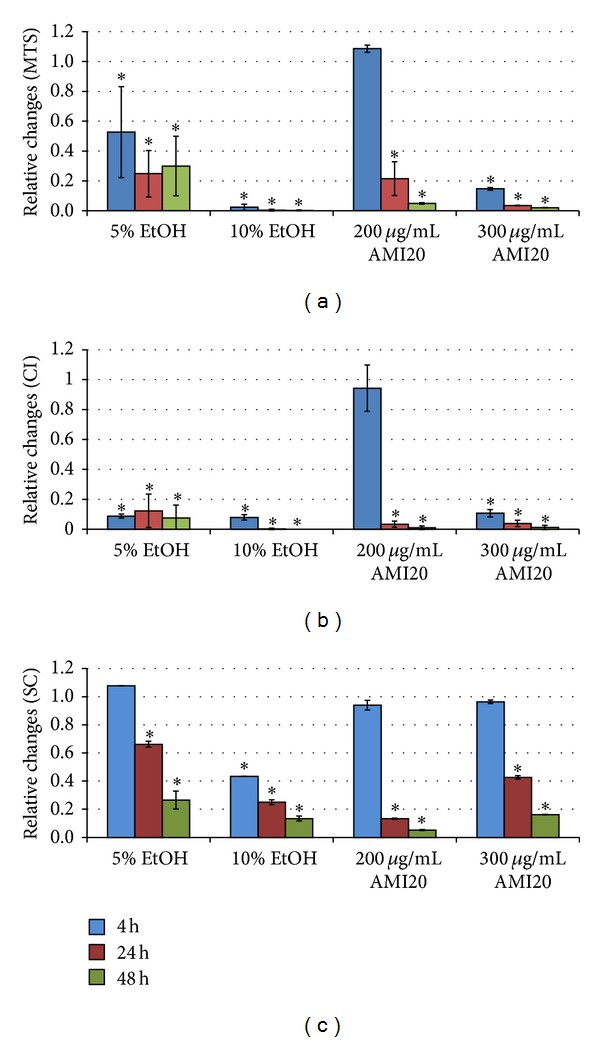
EAhy926 cells treated for 4 h, 24 h, and 48 h with different concentrations of ethanol (EtOH) and 20 nm amine polystyrene particles (AMI20) assessed by formazan bioreduction (MTS), according to cell index changes in the *xCELLigence* system (CI), and as stable cells (SC) according to image analysis by Cell-IQ software (*n* = 3). Changes are normalized to untreated controls as 1 and significant changes are (*P* < 0.05) indicated by asterisk.

To verify whether all systems identified toxicity of conventional substances or of particles that did not show obvious interference with assay systems, cells were exposed to different concentrations of EtOH and 20 nm AMI particles. While no significant reduction in viability was seen in the MTS, *xCELLigence* system, and Cell-IQ Analyzer after exposure to 2.5% ethanol, 5% and 10% EtOH reduced viability dramatically (Figure 2).

Upon exposure to 20 nm AMI, all systems showed concordant findings with no decrease in concentration of 100 *μ*g/mL but strong decreases after exposure to 200 *μ*g/mL and 300 *μ*g/mL (Figure 2). Decreases of stable cells in Cell-IQ after exposure to 200 *μ*g/mL 20 nm AMI particles were more pronounced than those after 300 *μ*g/mL. Inspection of the cultures treated with 300 *μ*g/mL AMI particles showed that they contained much cell debris, which apparently interfered with the classification process.

**Figure 3:**
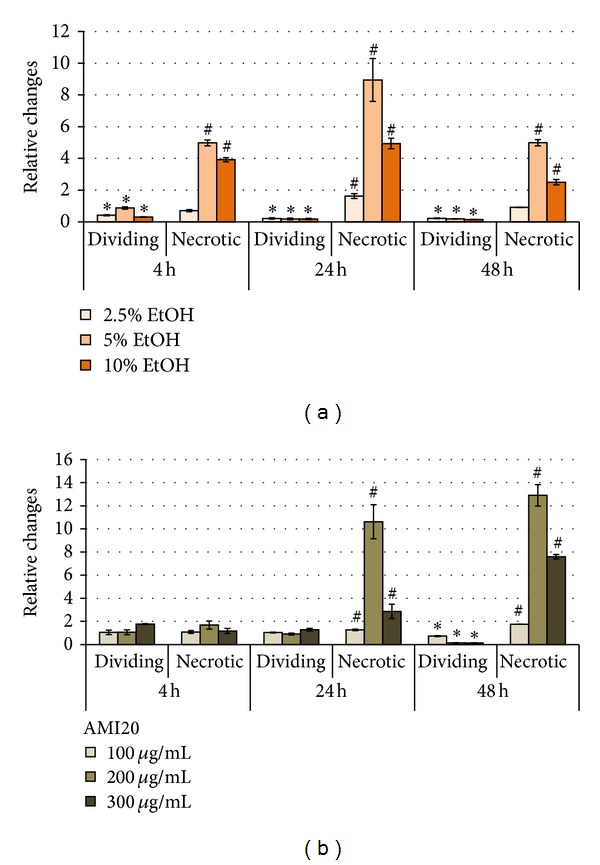
Cell-IQ software quantification of dividing and necrotic EAhy926 cells exposed to different concentrations of ethanol (EtOH (a)) and 20 nm amine polystyrene particles (AMI20 (b)). Changes are normalized to untreated controls as 1. Significant (*P* < 0.05) decreases are marked by asterisk and significant increases are indicated by hatch.

When changes in Cell-IQ were displayed as dividing and necrotic cells, it is obvious that the pattern compared to untreated cells was already altered after exposure to 2.5% EtOH at all time points (Figure 3(a)). The fraction of dividing cells was decreased and that of necrotic cells increased. At concentrations of AMI particles, which did not reduce the fraction of stable cells (100 *μ*g/mL), changes in the fraction of dividing and necrotic cells compared to untreated controls were obvious already after 24 h and 48 h of exposure.

With CNTs, differences between the effects of 25, 50, and 100 *μ*g/mL were relatively small. Therefore, in the following, the effects of 100 *μ*g/mL are described in more detail, while effects of lower concentrations will only be summarized in the final overview.

### 3.4. Cytotoxicity of CNTs in the MTS Assay

**Figure 4:**
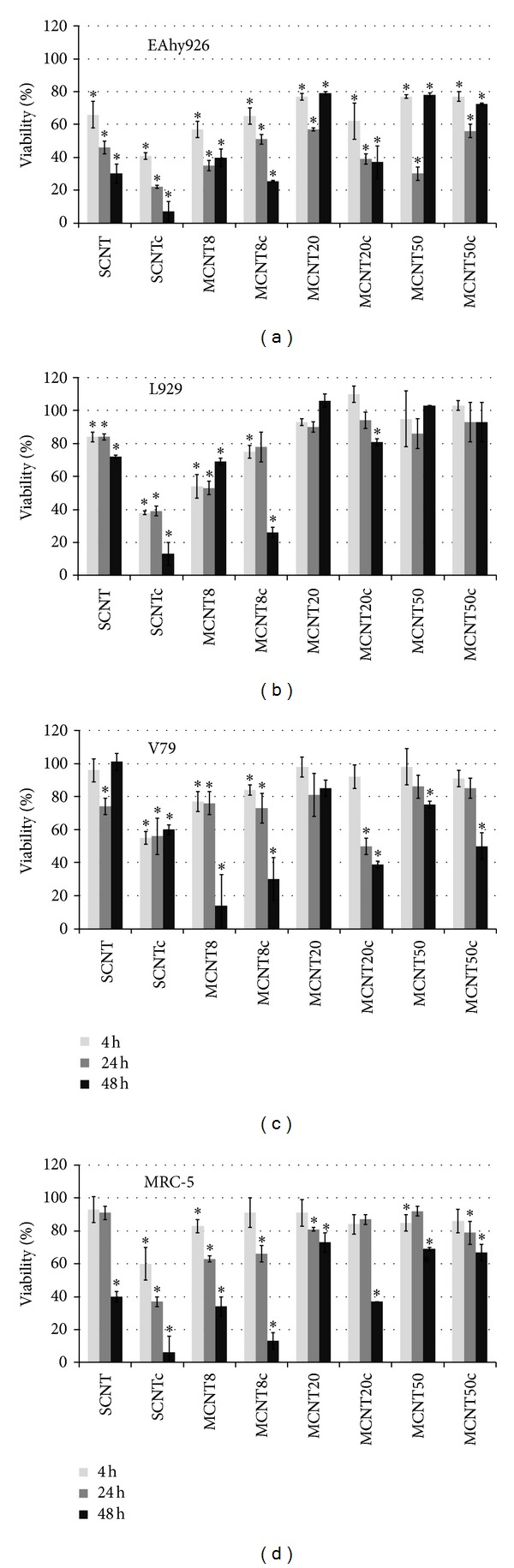
Viability according to formazan bioreduction (MTS assay) in different cell lines after exposure to CNTs (*n* = 4). Untreated cells are set as 100%. Significant changes are marked by asterisk.

Decreases in viability were more pronounced for SCNTc, MCNT8, and MCNT8c in all cell types. After 4 h at a concentration of 100 *μ*g/mL the following changes were observed (Figure 4): all CNTs produced significant decreases in viability of EAhy926 cells and only CNTs with diameters <8 nm induced significant decreases in L929 cells. SCNTc, MCNT8, and MCNT8c induced significant decreases in V79 cells and SCNTc, MCNT8, and MCNT50 in MRC-5 cells. After 24 h, incubation with SCNT and MCNT20c also decreased viability in V79 cells and MCNT8c, MCNT20, and MCNT50c in MRC-5 cells. After 48 h, with the exception of MCNT20, MCNT50, MCNT50c in L929 and MCNT20 in V79 cells, all CNTs caused significant decreases in viability.

### 3.5. Cytotoxicity by *xCELLigence* RTCA

**Figure 5:**
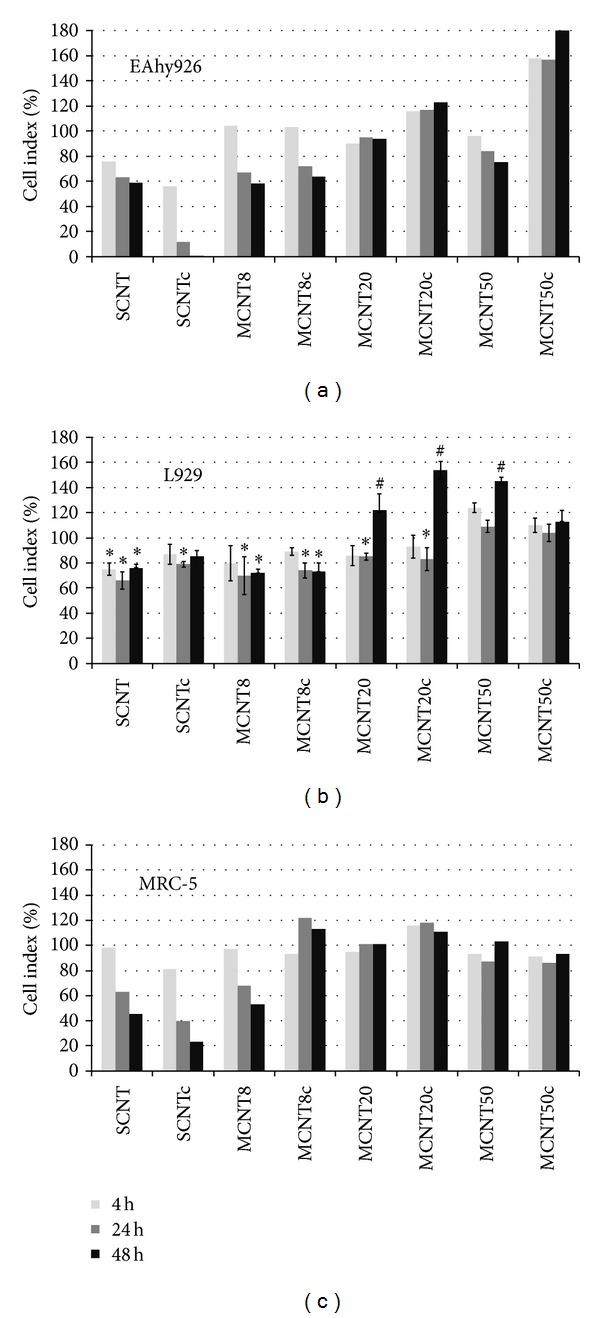
Changes in cell index assessed by *xCELLigence* system in different cell lines after exposure to CNTs normalized to untreated cells as 100% (L929, *n* = 3; other cell lines *n* = 2). Significant changes are marked by asterisk and increases by hatch.

After 4 h, the relative cell index of EAhy926 cells was strongly decreased (<80%) after incubation with SCNT and SCNTc. After 24 h also incubation with MCNT8, MCNT8c, and MCNT50 showed pronounced viability loss (Figure 5). After 24 h, viability of L929 cells was significantly reduced after incubation with MCNT20c. Transient decrease of viability was seen for MCNT20. The cytotoxicity pattern of MRC-5 cells was very similar to that of EAhy926 cells with prominent decreases for SCNT, SCNTc, and MCNT8 and lower cytotoxicity of the remaining CNTs. Differences between 24 h and 48 h of exposure were minimal.

### 3.6. Cytotoxicity by Cell-IQ Analyzer

**Figure 6:**
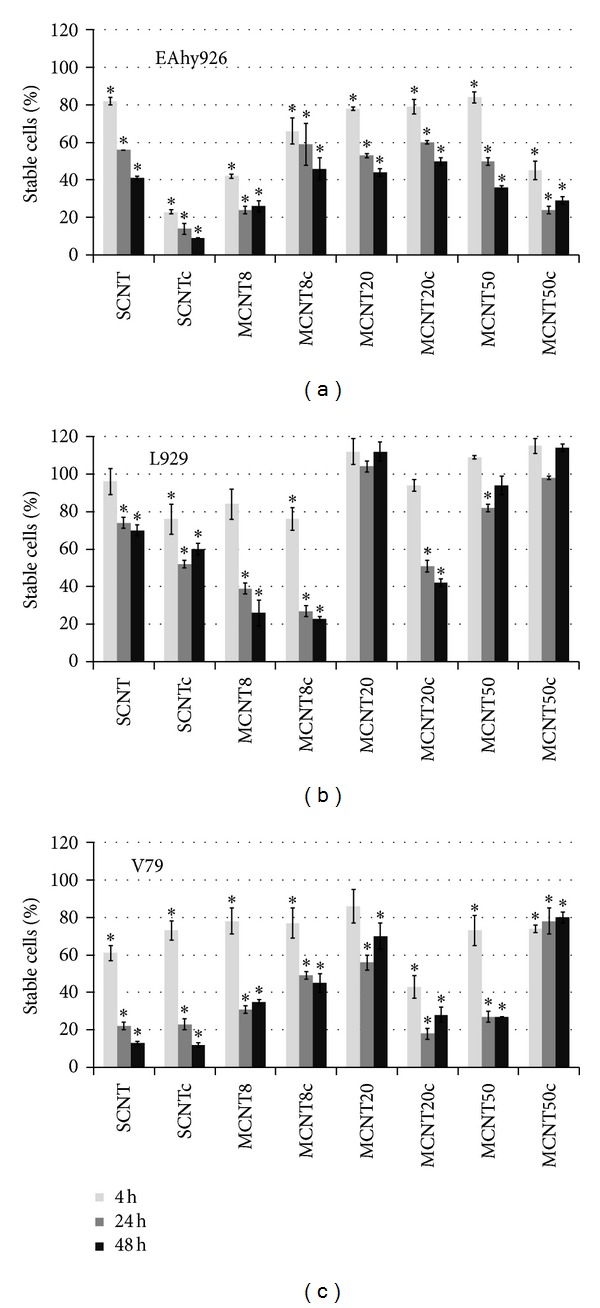
Fraction of stable cells according to Cell-IQ analysis in different cell lines exposed to CNTs (*n* = 4). Untreated cells are set as 100%. Significant changes are marked by asterisk.

Stable cells after incubation of EAhy926 cells with all CNTs for 4 h at a concentration of 100 *μ*g/mL were significantly decreased (Figure 6). In L929 cells, SCNTc and MCNT8c caused significant decreases after 4 h, while decreases for SCNT, MCNT8, and MCNT20c were significant only after 24 h. A transient decrease in viability after 24 h was observed after incubation with MCNT50. Viability of V79 was decreased for incubations with all CNTs, except MCNT20, after 4 h.

**Figure 7:**
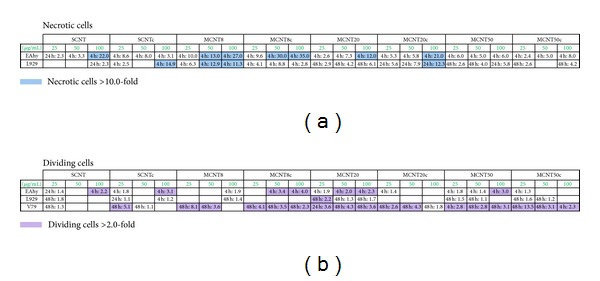
Detailed analysis of cellular reaction to CNTs according to Cell-IQ analysis software (*n* = 4). Necrotic cells (a) and dividing cells (b).

Based on earlier findings [15], disruption of membrane integrity leading to necrosis was identified as major cytotoxic mechanism of CNTs and, therefore, the contribution of necrotic cells was analysed in EAhy926, L929, and V79 cells. While no necrotic cells could be identified in V79 cultures, strong increases in necrotic EAhy926 cells were identified after CNT exposure (Figure 7(a)). L929 cells reacted with lower increases in necrotic cells to stimulation with CNTs. While all CNTs increased the rate of necrotic cells, most dramatic increases were seen for MCNT8, MCNT8c in EAhy926 cells (maximum 35-fold) and for SCNTc (maximum 14.9-fold) in L929 cells. Based on an arbitrary threshold set at a 10-fold increase of necrotic cells, CNTs < 8 nm induced slightly more often (in 5/24 exposures of EAhy926 cells and 3/24 exposures of L929 cells) necrosis than thick >20 nm CNTs (2/24 exposures of EAhy926 cells and 1/24 exposures of L929 cells). The strongest induction of necrosis was seen in EAhy926 cells and in L929 for thin CNTs after 4 h. Thick CNT induced necrosis in EAhy926 cells after 4 h and in L929 after longer incubation times. Carboxylation did not have an obvious influence on induction of necrosis.

Dividing cells were identified as potential reaction to CNT exposure. Increases in the fraction of proliferating cells were observed in EAhy926 cells, but rarely in L929 cells (Figure 7(b)). Increases in EAhy926 cells occurred after 4 h of exposure. Proliferation of V79 cells was markedly increased upon stimulation with all CNTs, except SCNT. This increase was usually obvious only after 48 h. In V79 cells, CNTs <8 nm induced proliferation to a lower extent than CNTs >20 nm 6/24 versus 11/24 exposures. Carboxylation did not appear to have an obvious influence on induction of proliferation.

### 3.7. Comparison of Cytotoxic Reaction in the Different Techniques

**Figure 8:**
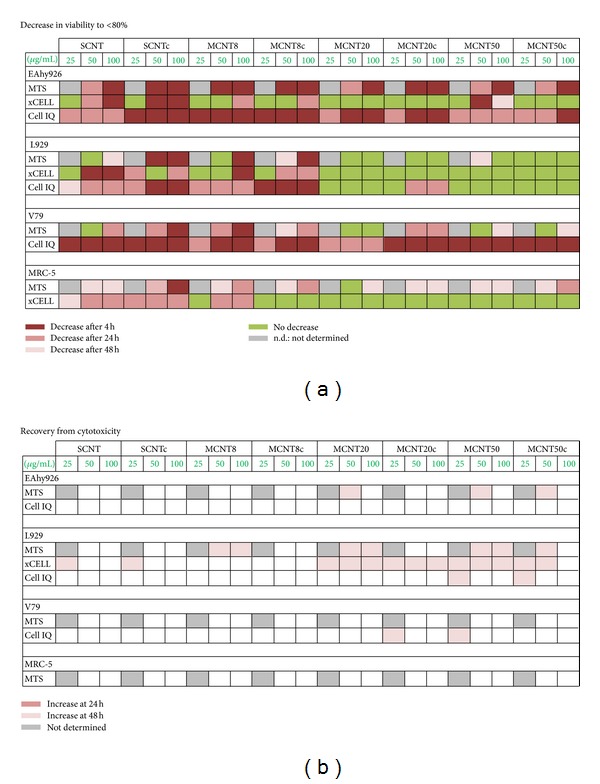
Comparison of detection systems regarding decrease of viability to <80% (a) and significant increases compared to viability at 4 h (recovery (b)). Higher sensitivity of cells and systems is indicated in deeper shades of red color, while exposures with lack of cytotoxicity are indicated in green.

Decreases of viability to <80% were used for comparison of the three techniques because, independent of the technique, such decreases were significant. In general, *xCELLigence* RTCA data produced a lower number of CNT exposures with decreases <80% (Figure 8(a)). By contrast, this system was more sensitive for detection of EtOH cytotoxicity (Figure 2). Cell-IQ and MTS analysis were similarly sensitive for cytotoxicity of CNTs in EAhy926 and L929 cells, while more CNTs were classified as cytotoxic by Cell-IQ than by MTS in V79 cells. Lower decreases in stable cells were recorded in Cell-IQ measurements after exposure to EtOH than in both other screening systems. When changes in dividing and necrotic cells were included in the evaluation, automated microscopy identified cytotoxicity at lower concentrations of EtOH and AMI particles than the other techniques.

Cell lines showed different sensitivities to the cytotoxic effects of CNTs. According to MTS, all CNTs induced decreases to <80% viability after 4 h in EAhy926 cells, while only SCNTc, MCNT8, and MCNT8c produced this decrease in L929 cells. SCNTc and MCNT8 decreased viability to <80% in V79 cells and SCNTc in MRC-5 cells. The *xCELLigence* RTCA identified SCNT, SCNTc, and MCNT50 as cytotoxic according to the definition mentioned above in EAhy926 cells and SCNT and MCNT8 in L929 cells. In Cell-IQ analysis all CNTs, except SCNT and MCNT50, were identified as cytotoxic in EAhy926 cells, SCNTc and MCNT8c in L929 cells, and all CNTs, except MCNT20, in V79 cells.

Decrease of cell viability to <80% after 4 h was induced by thin (<8 nm) CNTs more often than thick (>20 nm) CNTs. Out of the 24 exposure conditions of EAhy926 cells with different concentrations of CNTs, according to MTS assay, 7 (thin CNTs) versus 5 (thick CNTs) exposures reduced viability after 4 h. The corresponding ratios in *xCELLigence* RTCA were 2/24 for thin and 1/24 for thick CNTs and in Cell-IQ analysis 9/24 for thin and 5/24 for thick CNTs. In L929 cells 4/24 exposures to thin CNTs and 0/24 exposures to thick CNTs decreased viability according to MTS and Cell-IQ analyses. The corresponding ratios in *xCELLigence* RTCA were 3/24 for thin CNTs and 0/24 for thick CNTs. In the MTS assay 2/24 exposures to thin CNTs versus 0/24 exposures to thick CNTs decreased viability in V79 cells. Viability decreases according to Cell-IQ analysis (10/24 for thin CNTs and 9/24 for thick CNTs) were roughly similar. Only SCNTc reduced viability in MRC-5 cells according to the MTS assay and no CNTs decreased viability in *xCELLigence* RTCA.

Adaptation to the action or recovery from cytotoxicity was studied by comparing viability rates at 4 h, 24 h, and 48 h of exposure and look at time-dependent increases. The continuous monitoring by *xCELLigence* RTCA and Cell-IQ Analyzer allows the evaluation of additional time points. This, however, appeared not to be necessary because the shape of the growth curves did not reveal changes that were not identified also by comparison of viability at 4 h, 24 h, and 48 h. Increases in viability were usually observed at 48 h and predominantly in L929 cells (Figure 7(b)). *xCELLigence* RTCA identified a greater number of cellular recoveries than MTS and Cell-IQ Analyzer.

## 4. Discussion

This study shows that cytotoxicity of CNTs can be assessed by formazan bioreduction (MTS assay), impedance measurements (*xCELLigence* RTCA), and automatic microscopy (Cell-IQ Analyzer) with different sensitivity. All assays qualified thin (<8 nm) CNTs as more cytotoxic than thick (>20 nm) CNTs. This classification was most obvious in L929 cells and less distinct in the other cell lines and clearer in the MTS assay and Cell-IQ Analyzer than with *xCELLigence*. The degree of cytotoxic damage was higher in EAhy926 cells than in the other cell lines. Analysis by Cell-IQ Analyzer revealed a higher degree of induction of necrosis by thin CNTs and an increased proliferation rate upon incubation with thick CNTs. Time-dependent recovery to the cytotoxic action of CNTs was more pronounced for thick than for thin CNTs. A cell-specific reaction pattern to CNTs could be discerned: EAhy926 cells showed relatively high rates of necrosis, moderate proliferation rates, and little recovery from cytotoxicity. L929 cells, by contrast, showed moderate increases in the rate of necrotic and dividing cells but better recovery from CNT cytotoxicity than the other cell lines. In V79 cells, no necrotic cells were identified, proliferation was high, and some recovery to damage by CNTs was observed.

When selecting a screening method for the evaluation of NPs, label-free techniques may appear a good choice since interference with assay compounds can be prevented. The suitability of the label-free methods used in this study will be discussed regarding two aspects: problems inherent to the technology and specific problems with CNTs and not encountered with ethanol and polystyrene particles.

Limitations of the label-free methodologies used in this study were linked to the cells used. Measured impedance in the *xCELLigence* system depends on cell-substrate adherence, cell shape and volume, and cell-cell-interactions. While MRC-5 fibroblasts and endothelial EAhy926 cells reached the required CI of 1, DMBM-2 macrophages, apparently, showed too little adherence to the substrate and the CI was below 0.5. Mouse L929 fibroblasts also needed more than 24 h to reach the required CI value. Epithelial cells forming tight junctions appear to reach higher CI values than fibroblasts. Caco-2 cells reached a CI of 5 [27], HUVEC of 6 [28], while diploid fetal fibroblasts reached 3.5 [29].

Ethanol showed a stronger effect in *xCELLigence* than in the other two detection systems. Differences in cytotoxic effects can be explained by different degrees of confluence because confluent cells are more resistant to toxic damage than cells in the log phase [30, 31]. Such differences are unlikely to play a role for the different sensitivity of EAhy926 cells to ethanol in this study because cell densities in *xCELLigence* were not lower than those used for MTS and Cell-IQ experiments (Table 2). The higher sensitivity, however, could be due to slower growth in the E-plates and could also explain the approximately three times higher sensitivity of CI measurements by *xCELLigence* than viability in the WST-1 assay upon exposure to various cytostatic drugs [32]. After testing a panel of 21 conventional compounds, Atienzar et al. concluded that a CI decrease was not always associated with cytotoxicity effects and that confounding factors may affect the analysis [33]. *xCELLigence* has also been evaluated for the toxicity testing of several types of NPs and similar sensitivity of impedance measurements and MTT were seen in RAW 264.7 macrophages exposed to 20 nm SiO_2_ particles [34]. Testing of 11 inorganic nanomaterials by MTT assay and impedance measurements in 16HBE 140-cells identified ZnO, Mn_2_O_3_, and Ag NPs as most cytotoxic in both systems [35]. Gadolinium NPs embedded in polysiloxane shell caused reduction in viability of MDA-MB 231 cells in *xCELLigence* and MTT assays but decreases were visible much later in the *xCELLigence* than in the MTT assay [36]. A decreased sensitivity of *xCELLigence* to the cytotoxic action of CNTs compared to the other techniques was also observed in this study. Precipitation of CNTs on the electrode mimicking a higher coverage with cells or interference with the electric current used for impedance measurement could be reasons for the lower cytotoxic signal in this system.

Quality of analysis by Cell-IQ software was influenced by cell adhesion, cell size/shape, and cell deterioration. Loosely adherent or spindle-shaped cells posed problems and also the identification of apoptotic cells was not possible in this study. The formation of cell debris, seen after exposure of EAhy926 cells to 20 nm AMI particles, decreased the quality of cell classification. Label-free automated microscopy with noncommercial systems has already been used in compound screening [37]. Cytotoxic effects of plant extracts to Ehrlich's lymphoma ascites (ELA) cancer cells have also been detected by automated microscopy and validated by manual analysis [38]. The possibility to assess cytotoxicity based on image analysis has been validated with lactate dehydrogenase release and life/dead staining [39]. This study shows that the system could be suitable for assessment of colored NPs, such as CNTs, because cell classification by Cell-IQ analysis software was not impaired. Inclusion of dividing and necrotic cells increased the sensitivity of cytotoxicity screening.

One of the major advantages of continuous detection is the possibility to identify transient decreases in viability. Such changes are important, since they indicate cellular adaptation mechanisms. While linear decreases in viability were reported, for instance, for DMSO [20] and for PAMAM dendrimers [40], recovery from NP damage was seen after exposure to gold NPs [41] and to 50 nm silica particles [42]. Testing of five types of NPs in a panel of cell lines, Díaz et al. identified recovery, usually after 48 h of exposure, to various magnetic NPs [43]. Adaptation on the cellular level appears also likely for CNTs since the relative decrease in cell numbers was more pronounced after 7 days than after 15 and 28 days of exposure [44]. Although not all time points were evaluated, cellular recovery in this study was mainly identified in *xCELLigence* and Cell-IQ exposures. It is not clear why, despite the absence of the analysis of additional time points, continuous monitoring techniques identified recovery while endpoint cytotoxicity screening by MTS did not.

## 5. Conclusions

Label-free techniques cannot be used as universally as MTS for cytotoxicity screening. If automated microscopy can be used, however, further analysis of the mode of toxic action (cell division, necrosis, etc.) is advantageous. Limitations were due to specific requirements for the cells used in both systems. On the other hand, precipitated NPs can interfere with impedance measurements and lead to underestimation of their cytotoxic potential.

## Figures and Tables

**Table 1:** Characterization of CNTs suspended in DMEM + 10% FBS by Dynamic Light Scattering and Laser Doppler Velocimetry (DLS/LDV) and transmission electron microscopy (TEM).

Sample	DLS/LDV data		TEM data			
CNT	Hydrodynamic size (nm)	*ζ* (mV)	Diameter of single CNTs (nm)	Length of single CNTs (nm)	Diameter of CNT bundles (nm)	Length of CNT bundles (nm)
SCNT	16.4	−9.72	~2 nm	n.a.	28.3 ± 10.6	543 ± 60.8
SCNTc	15.7	−8.1	~2 nm	n.a.	62.5 ± 41.9	816 ± 275.4
MCNT8	26.8	−6.96	4.7 ± 0.48	222 ± 126.2	n.a.	n.a.
MCNT8c	16.3	−9.64	4.2 ± 0.8	217 ± 117.9	24.3 ± 5.1	600 ± 282.8*
MCNT20	124.4	−9.78	18.9 ± 0.9	446 ± 77.9	n.a.	n.a.
MCNT20c	38.8	−10.3	15.3 ± 2.5	251 ± 94.4	n.a.	n.a.
MCNT50	51.9	−7.28	62.8 ± 5.7	355 ± 96.4	n.a.	n.a.
MCNT50c	50.4	−11.0	63.6 ± 11.3	392 ± 195.3	n.a.	n.a.

n.a.: not analysed.

*Fraction in bundles approx. 50%.

**Table 2:** Seeding densities for MTS, *xCELLigence*, and Cell-IQ system (cells/cm^2^).

	MTS	*xCELLigence*	Cell-IQ
EAhy926	30000	50000	50000
DMBM-2	93750	100000	100000
L929	37500	62500	25000
MRC-5	37500	62500	25000
V-79	33000	50000	25000
